# Supplementary material for: Understanding the Impact of Salt Stress on Plant Pathogens Through Phenotypic and Transcriptomic Analysis
Source: Plants (Basel). 2025 Jan 1;14(1):97. doi: 10.3390/plants14010097 (PMC11722782; doi:10.3390/plants14010097)
Supplement: Supplementary file 1 [file plants-14-00097-s001.zip › Supplementary_Figures_R1.pdf]

Supplementary Figures

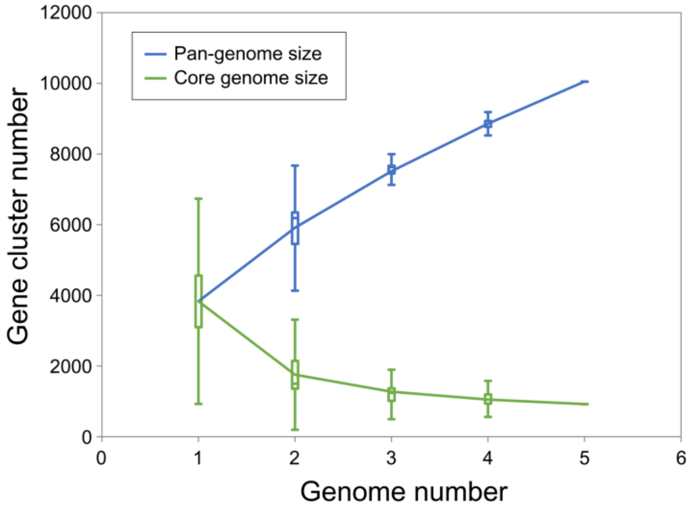

Figure S1. Pan-genome analysis of five plant pathogens. Pan- and core-genome profile curves of five plant pathogens (*B. gladioli* BSR3, *B. glumae* BGR1, *Pcc* PCC21, *R. solanacearum* GMI1000, and *Xoo* PXO99^{A}) indicate how pan-genome (blue) and core-genome (green) change as each genome is added in a random order. Curves are least-squares fits to a power law about average values. The *x*-axis represents the number of plant pathogens considered, while the *y*-axis indicates the number of genes.

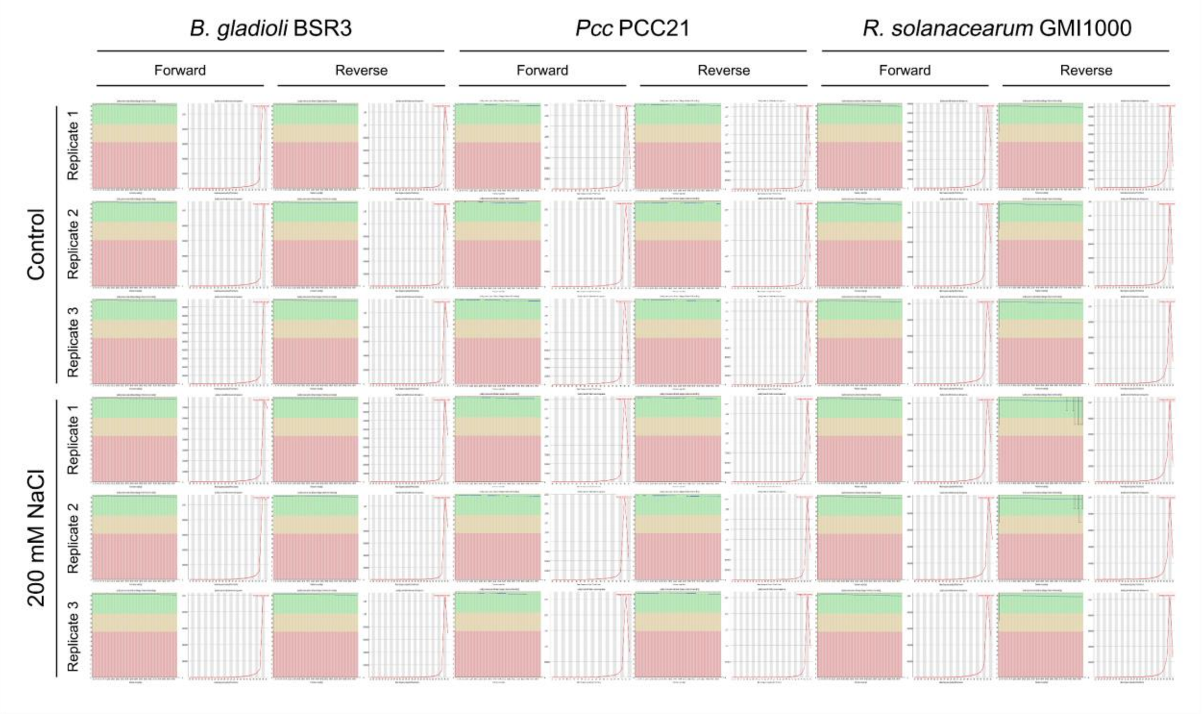

Figure S2. RNA-seq quality details. FastQC report illustrates the average quality scores across all bases of the paired-end datasets for 18 RNA-seq libraries in *B. gladioli*, *Pcc*, and *R. solanacearum*. The left panel represents the Phred quality scores for each nucleotide position, shown as a box and whisker plot. The central red line is the median value. The yellow box represents the inter-quartile range (25–75%). The right panel represents the quantitative distribution of reads by quality score.

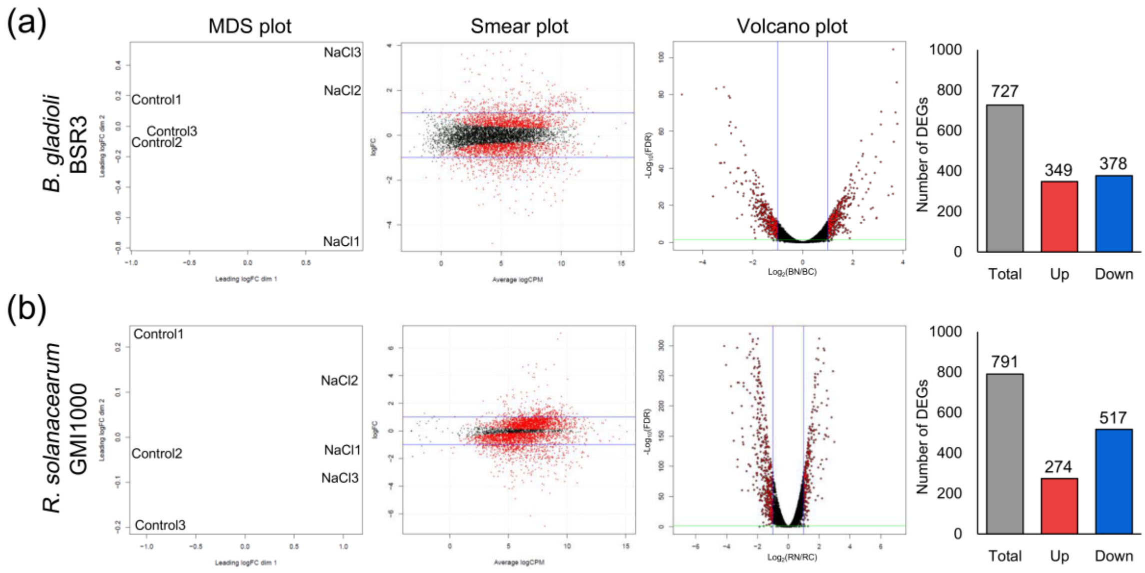

Figure S3. Differentially expressed genes (DEGs) analysis in salt-sensitive (**a**) *B. gladioli* and (**b**) *R. solanacearum*. The distances between RNA-seq libraries in response to 200 mM NaCl are visualized through a multidimensional scaling (MDS) plot. Smear plots depict the overall expression levels in salt treatment samples compared to control samples. In the volcano plot, each dot represents a gene, reflecting the $\log_2$(salt stress/control) and the –logFDR value. Red dots signify DEGs meeting the criteria of FDR < 0.05 and an absolute fold change > 2. The bar graph depicts the distribution of DEGs of plant pathogens under salt stress conditions, indicating the number of upregulated (red) and downregulated (blue) DEGs.
